# Efficient knockout of phytoene desaturase gene using CRISPR/Cas9 in melon

**DOI:** 10.1038/s41598-019-53710-4

**Published:** 2019-11-19

**Authors:** Isidre Hooghvorst, Camilo López-Cristoffanini, Salvador Nogués

**Affiliations:** 1 Departament de Biologia Evolutiva, Ecologia i Ciencies Ambientals, Secció de Fisiologia Vegetal, Universitat de Barcelona, 08028 Barcelona, España; 2 ROCALBA S.A., c/Barcelona 15, PO BOX 156, 17001 Girona, España

**Keywords:** Biological techniques, Genetic engineering

## Abstract

CRISPR/Cas9 system has been widely applied in many plant species to induce mutations in the genome for studying gene function and improving crops. However, to our knowledge, there is no report of CRISPR/Cas9-mediated genome editing in melon (*Cucumis melo*). In our study, phytoene desaturase gene of melon (*CmPDS*) was selected as target for the CRISPR/Cas9 system with two designed gRNAs, targeting exons 1 and 2. A construct (pHSE-CmPDS) carrying both gRNAs and the Cas9 protein was delivered by PEG-mediated transformation in protoplasts. Mutations were detected in protoplasts for both gRNAs. Subsequently, *Agrobacterium*-mediated transformation of cotyledonary explants was carried out, and fully albino and chimeric albino plants were successfully regenerated. A regeneration efficiency of 71% of transformed plants was achieved from cotyledonary explants, a 39% of genetic transformed plants were successful gene edited, and finally, a 42–45% of mutation rate was detected by Sanger analysis. In melon protoplasts and plants most mutations were substitutions (91%), followed by insertions (7%) and deletions (2%). We set up a CRISPR/Cas9-mediated genome editing protocol which is efficient and feasible in melon, generating multi-allelic mutations in both genomic target sites of the *CmPDS* gene showing an albino phenotype easily detectable after only few weeks after *Agrobacterium*-mediated transformation.

## Introduction

Genome editing tools have the potential to modify genomic sequences with accuracy. Some of these tools are: homologous recombination (HR), targeted induced local lesions in the genome (TILLING), zinc finger nucleases (ZFN), transcription activator-like effector nucleases (TALENs), or clustered regularly interspaced short palindromic repeats associated to nuclease Cas9 (CRISPR/Cas9). ZFN, TALENs and CRISPR/Cas9 are site-specific nucleases. The CRISPR/Cas9 genome editing tool was developed in 2013, and in comparison with other genome editing tools has better efficacy, efficiency, versatility and is simpler^1^.

CRISPR/Cas9 system cleaves a specific region of DNA by the Cas9 nuclease, which is guided by a 20-nt sequence named RNA-guide (gRNA). The association between Cas9 and gRNA, and the presence of a conserved protospacer-adjacent motif (PAM) downstream of the target DNA (typically NGG), allows a precise editing of DNA target sequences. The endonuclease domain induces DNA double-strand breaks (DSB), which can be repaired by either nonhomologous end-joining (NHEJ) or homology-directed repair (HDR) generating insertions and deletions events (INDELs) and substitutions^2,3^. The major uses of CRISPR/Cas9 in plants have been gene knockouts to elucidate the function of a target gene by gene mutation and transcriptional regulation^4^. This application allows genes function studies, to knock out genes that negatively affect food quality, to confer resistance to pathogens or divert metabolic flux away from valuable end-products^1^.

CRISPR/Cas9 system is theoretically applicable to all plant species, but many of them lack the experimental demonstration of its applicability. Since 2013, CRISPR/Cas9 has been applied in *Oryza sativa*^5^, *Arabidopsis thaliana*^6^, *Nicotiana benthamiana*^7^, *Solanum lycopersicum*^8^, *Zea mays*^9^ and soybean^10^, among other species. The strategy to mutate and knockout the phytoene desaturase gene (*PDS*) by CRISPR/Cas9 has been widely applied to quickly demonstrate the feasibility of CRISPR/Cas9 since its mutation causes photobleaching or albino phenotype. Through transient expression assays or transformation methods, CRISPR/Cas9 mutations have been demonstrated and studied in plants. Within the *Cucurbitaceae* family, this genome editing technique has only been reported as successfully applied in cucumber^11^ and watermelon^12,13^.

Melon (*Cucumis melo*) belongs to *Cucurbitaceae* family and is an important plant because of its specific biological properties and economic value of its fruit. In fact, 31 million tons of melon were produced worldwide in 2016^14^, being an important crop in Mediterranean and East Asian countries. The recent melon genome publication by Garcia-Mas *et al*.^15^ and the versatility of CRISPR/Cas9 allows to study and explore gene functions in melon. However, to our knowledge, CRISPR/Cas9 application in melon has not been reported. Therefore, the aim of this study was to demonstrate, for the first time, the applicability of CRISPR/Cas9 system on melon by performing a gene knockout of the melon phytoene desaturase gene (*CmPDS*) in protoplasts and plants.

## Results

### Target selection and vector construction for CRISPR/Cas9 system

**Figure 1:**
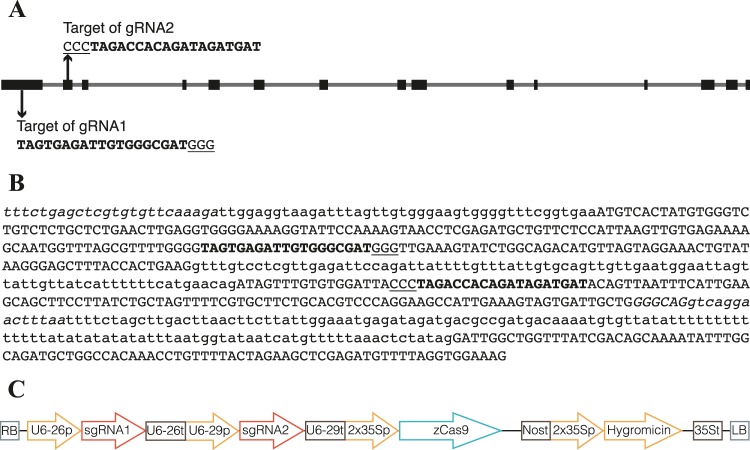
Schematic representations of the melon *CmPDS* target gene (MELO3C017772.2 in ICUGI data base), location of the gRNA1 and gRNA2, and the CRISPR/Cas9 vector. (**A**) Schematic representation of melon *CmPDS* gene with two target sites indicated as bold and PAM sequences as underlined. (**B**) Representation of *CmPDS* target sequences. Exons are shown as capital letters, introns as lowercase, gRNA1 and gRNA2 as bold, PAM sequences as underlined, and primers used for gene sequencing as italics. (**C**) Schematic representation of the CRISPR/Cas9 binary vector used for melon transformation. *Arabidopsis thaliana* promoters and terminators drive expression of gRNA1(AtU6–26p and AtU6–26t) and gRNA2 (AtU6–29p and AtU6–29t). The Cauliflower mosaic virus promoter (CaMV 35S) drives the expression of the Cas9 gene.

To test the efficacy of CRISPR/Cas9 system in melon, we chose to disrupt phytoene desaturase gene of *Cucumis melo* (CmPDS) which has a single copy, located in the chromosome 7, with reference MELO3C017772.2 in ICUGI data base. The genomic sequence of this reference is 10,443 bp in size, with 14 exons (Fig. 1A). Target sites in *CmPDS* were designed using Benchling^16^ and two target sites were selected: gRNA1 and gRNA2, in exon 1 and 2 respectively (Fig. 1A). Both gRNAs were cloned into one binary vector (pHSE-CmelPDS) carrying the promoter for the Cas9 gene (CaMV 35S), the Cas9 gene, AtU6–26p and AtU6–29p promoters and gRNA scaffold (Fig. 1C). Potential off-target sites were searched using CRISPR-OFFinder^17^, and no potential off-target were detected with 0 to 2 mismatches (Table S1)^18^.

### Targeted mutagenesis in melon protoplasts

CRISPR/Cas9 vector pHSE-CmelPDS was tested in protoplasts to validate the functionality of Cas9 via transient expression by PEG-mediated protoplast transfection. A total of 24 protoplast cell colonies were Sanger-sequenced. According to the number of mutated colonies detected, the target efficiency for pHSE-CmelPDS in melon protoplasts was 25% for both gRNAs. Moreover, most of the mutations analyzed were substitutions and two nucleotides insertions (Fig. 3 and Table S2). These results suggested that the vector was viable to be used in melon for plant regeneration.

### PDS-edited plants phenotype

**Figure 2:**
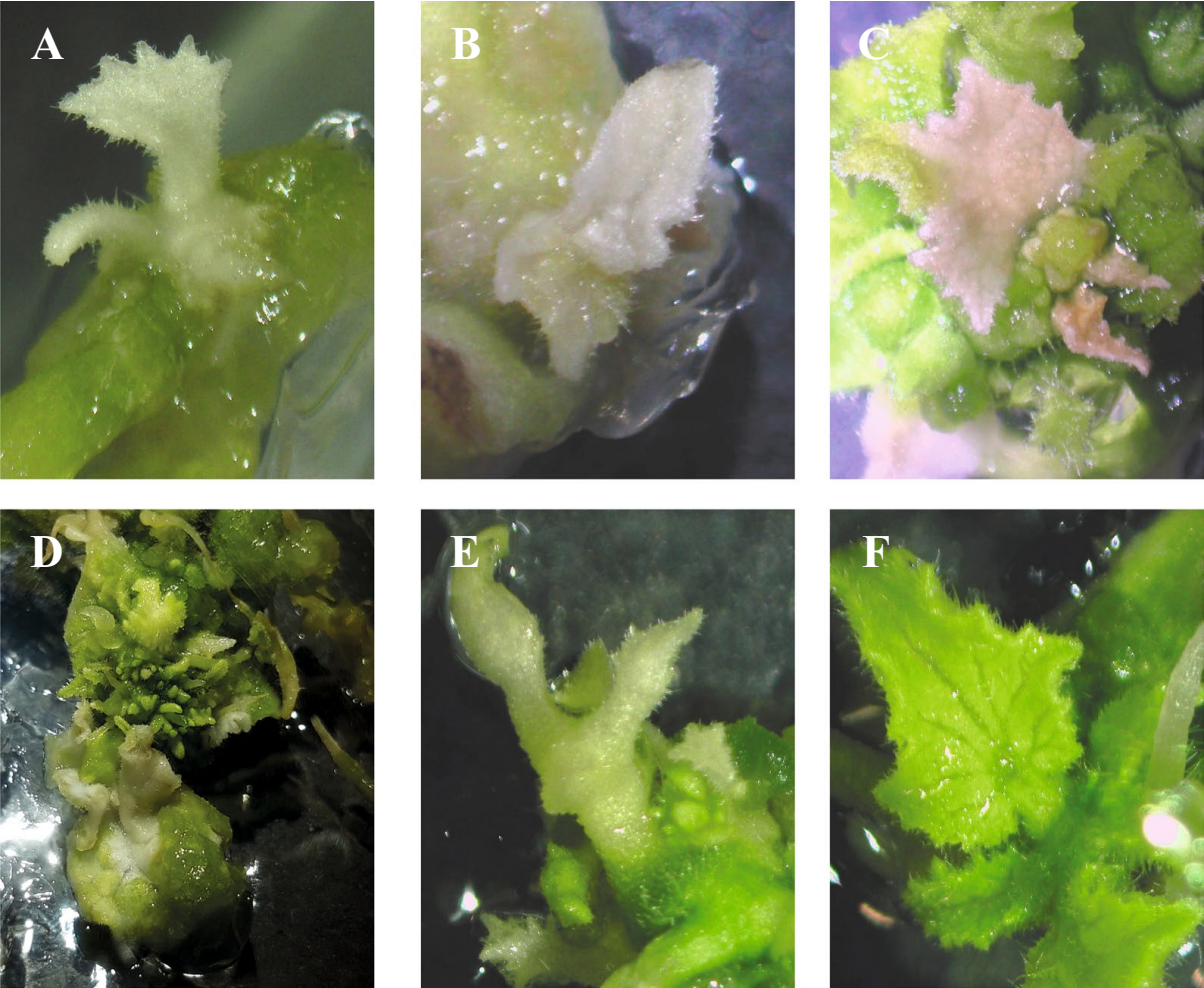
Phenotypic diversity of regenerated CRISPR/Cas9 mutated plants. (A,B) panels show fully albino and dwarf melon plants; (C–E) panels show chimeric albino plant presenting a mixture of green and white tissues; and (F) panel, shows a green WT regenerated plant.

Alteration of *CmPDS* gene expression function was manifested as albino and dwarf plants (Fig. 2). Some cotyledons only regenerated albino shoots, meanwhile others regenerated green shoots and after a while exhibited mosaicism or regenerated secondary albino shoots. Complete albino plants exhibited a high level of dwarfism, having a reduced leaf area, a growth lesser than 0.5 cm, and impossibility to perform *in vitro* propagation. A total of 77 plants were regenerated from 958 *Agrobacterium*-mediated transformed cotyledonary explants. The 22.07% of the regenerated plants showed albino phenotype, 4 complete albino plants and 13 chimeric albinism were detected. Albino plants did not survive more than 3 months after regeneration.

### Targeted mutagenesis in transgenic melon plants

**Figure 3:**
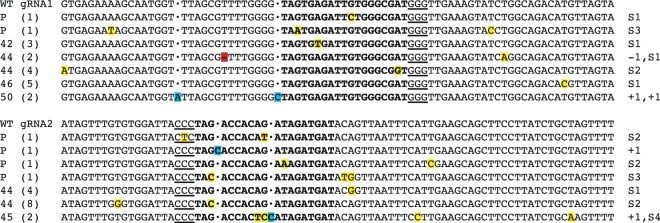
Targeted mutations detected in melon plants and protoplasts induced by CRISPR/Cas9 in each gRNA. Target sequences of *CmPDS* are bolded and the protospacer adjacent motif (PAM) in italics. Aligned sequence data is shown for four plants (number) and protoplasts (P). Number of colonies sequenced are indicated in the left between parentheses. Substitutions are highlighted in yellow; insertions are highlighted in blue, whereas a **·** is used in sequences with no insertion to maintain the reading frame; and, deletions are highlighted in red.

Polymerase chain reaction (PCR) was performed in genomic DNA of the regenerated plants to detect the presence of the transgene and to amplify the target gene sequences. Genomic DNA was extracted from 62 plants. A 239 bp fragment within the LB and RB regions was amplified to confirm the presence of transgene. The transformation efficiency on the regenerated plants was 71%. A 502 bp fragment of the *CmPDS* gene corresponding to the first and second exons together was amplified, and a mutation analysis was performed with 10 plants showing a clearly *PDS*-edited phenotype (Fig. 3). A variety of mutations was detected in the genotyped plants, including insertions, deletions and substitutions (Fig. 3 and Table S3). In 113 sequenced colonies from albino phenotype plants analyzing both gRNAs, the target mutation efficiency was 45% and 42% for gRNA1 and gRNA2, respectively. Up to 91% of mutations were substitution events, 7% were insertions, and 2% deletions. Further analysis of the *CmPDS* gene mutations revealed substitutions, insertions and deletion events upstream and downstream of the target region for both gRNAs. Multiple alleles were found in all albino and chimeric plants, showing a high level of chimerism for both target sequences (Table S3). *In silico* analysis of codon change due to substitutions in plants revealed that 28.56% of substitutions caused an introduction of a STOP codon, 60.71% caused a change in amino acid (AA) codon, and a 10.71% caused no AA codon change.

## Discussion

To date, CRISPR/Cas9 system has been applied for basic research and trait development in many plant species^19,20^. To our knowledge, this study reports for the first time gene editing by CRISPR/Cas9 in melon (*Cucumis melo*). We employed this system to target the *PDS* gene in melon, a key enzyme in the carotenoid biosynthesis pathway involved in at least 20 metabolic pathways, including the inhibition of many genes in carotenoid, chlorophyll, and GA biosynthesis pathways^21^. Successful disruption of *PDS* gene results in a generation of mutants expected to be photobleached or albino, which allows to phenotypically identify the feasibility of CRISPR/Cas9 in melon. Alteration of *PDS* was chosen as a visual marker to easily detect the CRISPR/Cas9 genome editing in melon. In order to improve our chances of success we designed two gRNAs and adapted an existing genetic transformation system in cucumber^22^. Chimeric plants, showing albino and green tissue in the same plant or leaf, and albino plants were regenerated from melon cotyledonary explants, as reported in many other species^23–27^. A total of 4 fully albino plants and 13 albino chimeric plants were regenerated due to the loss-of-function of *CmPDS* gene.

Most of the mutations analyzed in melon protoplasts were substitutions and two insertions were found. Mutations in transgenic melon plants were mostly substitutions, with some deletion and insertion events. Although INDELs are the most common mutations induced by NHEJ, other authors have found similar results concerning the high level of substitutions induced by CRISPR/Cas9. For example, in soybean protoplasts and plants, a high level of substitutions were analyzed in the mutations events^28^; in protoplasts of cotton, only substitutions were detected^29^; in cassava plants, substitutions occurred more frequently than INDELs mutations^24^; and, in rice, the 25–45% of the analyzed mutations were substitutions^30^.

*Agrobacterium*-mediated transformation is the only reported method for genetic transformation in melon. One of the major issues when transforming species of the *Cucurbitacea* family is the occurrence of “escapes”, which are non-transgenic well-developed shoots regenerated into selective media. In melon, the regeneration rates from cotyledonary explants are usually high (40–70%), nevertheless, transformation rates of the regenerated shoots are usually very low, and is highly genotypic dependent^22,31^. Melon, of the *Cucurbitacea* family, is a recalcitrant crop for transformation^31^. In our study, 8% of the cotyledonary explants regenerated shoots, among which a high percentage (71%) proved to be transgenics. Although we did not find biallelic mutations in melon plants and albino plants failed to grow *in vitro*, mutations should be stably inherited in the T1 and T2 generations^32^. The lethal phenotype of PDS mutant plants could be alleviated by optimizing the medium. The use of an additional medium to grow albino plants, increasing sugar composition or increasing cytokinin proportions could allow the survival of albino plants. Furthermore, plant regeneration from cotyledonary explants can induce endoduplication phenomenon that generate tetraploid regenerants^33^. In this experiment we did not analyze the ploidy of the regenerated albino plants because majority of albino plants were also dwarf hence plant material availability was low.

The pHSE-CmelPDS vector contained the Cas9, a gRNA scaffold and both gRNA guides. Promoters from the cauliflower mosaic virus (CaMV35S) and *Arabidopsis thaliana* (U6 and U3), were used for driving the constitutive expression of Cas9 and both sgRNA (gRNA1 and gRNA2), respectively. Within dicotyledons, when CaMV35S promotor is used the mutation rate depends on the species, from 20 to 100%^26,34^. High transcription levels of Cas9 protein and sgRNA are essential for the activity of the CRISPR/Cas9 system^35^. The expression driver of Cas9 gene is an important factor that can increase the mutation frequency, and the use of a endogenous promoter ensures a high level of transgene expression and allows to increase mutation efficiencies 2 to 7 fold, as showed in soybean and liverwort^28,36^. The endogenous U6 promoter for sgRNA expression has a key role in the CRISPR/Cas9 efficiency too^37^. The regeneration efficiency of mutant plants from cotyledonary explants (1.8%) was very similar to those reported in watermelon^12^. The mutation rate of the sequenced colonies was 42–45% in the analyzed transgenic plants, which is lower than the ones found in other dicotyledons. In protoplast transfected cells, mutation was successful despite low edition frequencies, 25% for each gRNA. In comparison to other species this value is low and could be due either to low transfection efficiency or to a lack of CRISPR/Cas9 editing activity. It is possible that the fact that the promoters used were not endogenous is the cause of the efficiency drop of the CRISPR/Cas9 system. Overall, the use of endogenous promoters for Cas9 and sgRNA expression is the best way to increase the CRISPR/Cas9 efficiency in melon and other cucurbits.

Efficient gene editing in melon presents the possibility to study new gene functions for basic research, and new opportunities for melon productivity by improving biotic stress, melon production and the post-harvest utilization. Towards this, we demonstrated the occurrence of targeted mutagenesis with the CRISPR/Cas9 system in melon protoplasts and plants. The *CmPDS* knockout system described generates easily detectable albino and dwarf plants and mutation events in a frame of 6–8 weeks. Therefore, it provides a valuable method to facilitate rapid assessment and optimization of CRISPR/Cas9 and other genome-editing technologies in melon.

## Materials and Methods

### Vector construction

Construct for constitutive expression of Cas9 was done following the protocol of Xing *et al*.^38^. The binary vectors pHSE401 and pCBC-DT1T2 (Addgene plasmids #62201 and #50590, respectively) were a gift from Qi-Jun Chen. For the assembly of two gRNAs into pHSE401, a four-primer mixture with DT1-F0-PDS/DT2-R0-PDS and DT1-BsF-PDS/DT2-BsR-PDS in a proportion 1:20 (Table S5), were used for PCR amplification along with pCBC-DT1T2 and Phusion High-Fidelity DNA Polymerase (NEB) following the manufacturer’s recommendations. PCR protocol was 94 °C for 2 min, followed by 30 cycles of 94 °C for 15 s, 60 °C for 30 s, and 68 °C for 1 min and a final extension at 68 °C for 5 min. PCR product DT1T2-PCR (626 bp) was separated on 2% agarose gel and agarose purified with PureLink Quick Gel Extraction Kit (Invitrogen). Then, DT1T2-PCR product was assembled into pHSE401 by Golden Gate cloning method, using *Bsa*I and T4 Ligase (NEB) following the manufacturer’s recommendations. The binary vector constructed, named as pHSE-CmelPDS (Fig. 1C), containing both guides, gRNA1 and gRNA2, was used to transform NEB 5-alpha Competent *E. coli* (High Efficiency; NEB). Positive clones were confirmed by Sanger sequencing, plasmids were isolated using GeneJET Plasmid Miniprep Kit (Thermo Scientific) and finally transformed into *Agrobacterium tumefaciens* EHA105.

### Extraction of melon protoplast and its transformation

Melon protoplasts were extracted and transfected following the protocol from Yoo *et al*.^39^ with minor modifications. 10–15-days old melon cotyledons were chopped with a razor blade and digested with 10 mL of enzyme solution (20 mM MES, 1.5% cellulase R10, 0.4% macerozyme R10, 0.4 M mannitol, 20 mM KCl and pH 5.7) and incubated in an orbital shaker for 3 h at 24 °C and 70 rpm. Then, the protoplast solution was collected on ice into a 15 mL tube and diluted up to 10 mL with W5 solution (2 mM MES, 0.5 M mannitol, 20 mM KCl and pH 5.7). The protoplast solution was filtered through a 75-µm mesh, and protoplasts were collected by centrifuging at 100 g for 5 min at 4 °C. The supernatant was discarded and protoplasts were resuspended up to 10 mL of W5. Protoplasts were kept on ice for 30 min, the supernatant was discarded, and the protoplast solution was adjusted to a concentration of 2·10^5^ protoplasts·mL^−1^ with the help of a hemocytometer with MMG solution (4 mM MES, 0.4 mannitol, 15 mM MgCl_2_ and pH 5.7). For the transformation, 10 µL of pHSE-CmelPDS vector (20–30 µg) were mixed with 100 µL of protoplasts (2·10^4^) and 110 µL of PEG-calcium transfection solution (40% PEG4000, 0.2 M mannitol, and 100 mM CaCl_2_), and incubated for 15 min at 25 °C in darkness. Transfection was stopped by adding 400 µL of W5 supplemented with 5 mg·L^−1^ hygromycin and cultured in darkness for 24 h at 24 °C. The transfected protoplasts were then collected for genomic DNA extraction.

### Agrobacterium-mediated transformation and plant regeneration

*Cucumis melo* var. *cantaloupensis* inbred line “Charentais” (provided by ROCALBA S.A.) was used as the source of explants for genetic transformation. Seed coats were removed, and seeds were surface-sterilized with a 20% sodium hypochlorite solution containing 8 drops·L^−1^ of Tween 20 for 10 min in agitation and rinsed two times with sterile distilled water. Seedlings were pre-cultured on MS medium supplemented with 3% sucrose for two days. Then, nodal cotyledons were used as explants after removing the embryo. Explants were vacuum-infiltrated with *Agrobacterium tumefaciens* EHA105 infection solution for 5 min twice and an additional 5 min in a shaker without vacuum. To prepare the *Agrobacterium tumefaciens* EHA105 infection solution, a single colony containing the selected binary CRISPR/Cas9 vector was picked and put in a 5 mL starter culture of LB containing 50 mg·L^−1^ Kanamycin and 100 mg·L^−1^ Rifampicin and cultured for 20 h at 24 °C at 130 rpm. Next, 1 mL of the starter culture was transferred to 45 mL LB culture containing Kanamycin and Rifampicin and cultured until OD_600_ reached 0.6. Once the *Agrobacterium* transformation was carried out, explants were transferred to co-cultivation medium consisting on MS medium supplemented with 3% sucrose and cultured 48 h at 24 °C in the dark. Afterward, explants were rinsed with antibiotic solution composed of 500 mg·L^−1^ Cefotaxime and 300 mg·L^−1^ Ticarcillin in distilled water. Then, the explants were transferred into selective medium which was MS supplemented with Sucrose 3%, 2.22 µM BAP, 0.48 µM IAA, 4 µM CuSO_4_·5H_2_O, 500 mg·L^−1^ Cefotaxime, 300 mg·L^−1^ Ticarcillin and 5 mg·L^−1^ hygromycin B. Every two weeks explants were transferred to fresh selective media. Regenerated plants were transferred to E20A^40^ media supplemented with 5 mg·L^−1^ hygromycin B. Explants on selective medium and plants were cultured under a 16/8 h light/dark photoperiod at 24 °C.

### Detection of transgene and CRISPR/Cas9 mutation

Genomic DNA of protoplasts and plants was extracted following the methodology of Doyle and Doyle^41^ with minor modifications. The transgene presence in plant (Fig. 1B) was confirmed by PCR using specific primers named pHSE401.SeqF and pHSE401.SeqR (Table S5). A fragment flanking gRNA1 and gRNA2 (846 bp) of the *CmPDS* gene from transgenic plants and transformed protoplasts was amplified using F-CmPDS and R-CmPDS primers, and the amplified PCR product was gel-purified and cloned into pCR-Blunt II-TOPO vector (Life Technologies). Colonies were Sanger-sequenced using M13F and M13R primers for detecting specific additions, deletions and substitutions. Mutation rate was calculated as the ratio of mutated clonal colonies versus total sequenced colonies.

## Supplementary information

Supplementary Data
