# Supplementary material for: Efficient knockout of phytoene desaturase gene using CRISPR/Cas9 in melon
Source: Sci Rep. 2019 Nov 19;9:17077. doi: 10.1038/s41598-019-53710-4 (PMC6863862; doi:10.1038/s41598-019-53710-4)
Supplement: Supplementary file 1 — Supplementary Data [file 41598_2019_53710_MOESM1_ESM.pdf]

Supplementary material

Efficient knockout of phytoene desaturase gene using CRISPR/Cas9 in melon

Isidre Hooghvorst[1,2], Camilo López-Cristoffanini[1], Salvador Nogués[1]

[1]Departament de Biologia Evolutiva, Ecologia i Ciencies Ambientals, Secció de Fisiologia Vegetal, Universitat de Barcelona, 08028 Barcelona, España

[2]ROCALBA S.A., c/Barcelona 15 PO BOX 156, 17001 Girona, España

***Correspondence:** Isidre Hooghvorst, email: isidrevander@gmail.com.

Table S1. Analysis of gRNA sequences used for CRISPR/Cas9 vector construction.

	Nucleotide sequence (5' - 3')	Exon and position	PAM	GC (%)	Mutation rate (%)	
					Protoplast	Plants
gRNA1	TAGTGAGATTGTGGGCGAT	Exon 1; 123-141	GGG	47.39	25	45
gRNA2	TAGACCACAGATAGATGAT	Exon 2; 21-39	GGG	36.84	25	42

Table S2. Summary of the alleles, number of colonies and frequency of each allele (%) found in protoplast cells PEG4000-mediated transformated with the CRISPR/Cas9 vector. i: insertions; s: substitution; WT: wild-type.

Alleles found	nº colonies	Frequency (%)
3s/2s	1	4.17
WT/1s	1	4.17
1s/1s	1	4.17
1s/WT	1	4.17
1s/1i	1	4.17
1s/1i	1	4.17
1s/3s	1	4.17
WT/WT	17	70.83

Table S3. Summary of the genotyping of ten plants with albino phenotype. Type of mutation of each allele found, number of colonies, frequency of each allele, edition profile of the plant and phenotypic appearance. d: deletions; i: insertions; s: substitution; WT: wild-type.

Plant number	Sequenced colonies	Type of mutation	Frequency (%)	Edition profile	Phenotypic appearance
33	2	3s/WT	22.22	Heterozygous	Chimeric
33	7	WT/WT	77.78		
40	7	1s/1s	77.78	Chimeric	Chimeric
40	1	1s/WT	11.11		
40	1	WT/WT	11.11		
42	3	1s/WT	23.08	Heterozygous	Chimeric
42	10	WT/WT	76.92		
44	2	1d1s/1s	13.33	Chimeric	Albino
44	4	2s/1s	26.67		
44	8	WT/2s	53.33		
44	1	WT/WT	6.67		
45	3	WT/1i4s	25.00	Chimeric	Albino
45	9	1s/WT	75.00		
46	5	1s/WT	33.33	Heterozygous	Albino
46	2	WT/2s	13.33		
46	2	WT/1s	13.33		
46	6	WT/WT	40.00		
49	3	WT/1s	27.27	Heterozygous	Chimeric
49	8	WT/WT	72.73		
50	10	1s/WT	83.33	Heterozygous	Chimeric
50	2	2i/WT	16.67		
59	6	1s/WT	75.00	Heterozygous	Chimeric
59	2	2i/WT	25.00		
60	7	1s/1s	77.78	Heterozygous	Chimeric
60	2	WT/WT	22.22		

Table S4. *In silico* analysis of CRISPR/Cas9 induced substitutions in plants. Nucleotide substitution could result in: introducing a STOP codon, introducing a new amino acid (AA) codon, or, no change in the amino acid codon.

Total number of substitutions	Substitutions causing STOP codon	Substitutions causing AA change	Substitutions causing no AA change
28	28.58%	60.71%	10.71%

Table S5. Primers used in this study to assemble two gRNAs in the pHSE401 vector, for transgene detection and for gene amplification and sequencing of *CmPDS* gene.

Primer name	Primer sequence (5' - 3')
Assembly of two gRNA	
DT1-BsF-PDS	ATATATGGTCTCGATTGCGAGATGCTGTTCTCCATTGTT
DT1-F0-PDS	TGAATGGAGAACAGCATCTCGGTTTTAGAGCTAGAAATAGC
DT2-R0-PDS	AACTAGACCACAGATAGATGATACAATCTCTTAGTCGACTCTAC
DT2-BsR-PDS	ATTATTGGTCTCGAAACTAGACCACAGATAGATGATC
Detection of transgene	
pHSE401.SeqF	TCTTCAAAAGTCCCACATCGC
pHSE401.SeqR	AACCCCAGAAATTGAACGCC
CmPDS gene amplification	
F-CmPDSgRNA1	CTGAGCTCGTGTGTTCAAAGA
R-CmPDSgRNA2	TTAAAGTTCCTGACCTGCCC
